# The olfactory-glymphatic syndrome: linking smell dysfunction, cognitive impairment and sleep disturbances in neuropathological disorders

**DOI:** 10.3389/fnins.2026.1897376

**Published:** 2026-08-12

**Authors:** William Thomas Phillips, Joyce Gensberg Schwartz

**Affiliations:** 1 Department of Radiology, UT Health San Antonio, San Antonio, TX, United States; 2 Department of Pathology, Methodist Hospital, San Antonio, TX, United States

**Keywords:** cognitive impairment, glymphatic dysfunction, metabolic syndrome, nasal turbinate vasodilatation, neurodegenerative disease, neuropathological disorders, olfactory dysfunction, sleep disturbances

## Abstract

Olfactory dysfunction is a common feature of several neuropathological disorders – including Alzheimer’s and Parkinson’s diseases – whose shared profile often includes glymphatic dysfunction, cognitive impairment, and sleep disturbances. Importantly, smell loss frequently precedes cognitive or motor symptoms by years, highlighting its potential as an early clinical marker. This article reviews mechanisms that may link olfactory dysfunction to disease development in a wide range of neuropathological diseases and advances a research-based hypothesis for a shared etiologic pathway underlying these overlapping symptoms. In particular, it examines metabolic syndrome as a possible upstream driver that promotes nasal turbinate vasodilation, disrupts nasal lymphatic drainage, and culminates in an olfactory-glymphatic syndrome. Integrating glymphatic imaging or assessment with olfactory testing, cognitive evaluation, and metabolic risk profiling – hypertension, diabetes mellitus, hyperlipidemia, and elevated body mass index – may enable earlier detection and open new avenues for prevention and novel therapeutic opportunities for neuropathological disorders.

## 1 Introduction

Neuropathological diseases represent a growing global health burden, affecting millions of individuals worldwide (Erkkinen et al., 2018). Traditional diagnostic approaches rely on the emergence of characteristic motor or cognitive symptoms which often manifest only after substantial, irreversible neuronal loss has occurred. Consequently, identifying reliable biomarkers that signal disease onset during prodromal stages is critical to enabling earlier therapeutic intervention and improving clinical outcomes.

Olfactory dysfunction – characterized by a reduced ability to detect, identify, or discriminate odors – is a common yet frequently overlooked early manifestation of an expanding range of neuropathological disorders (Dan et al., 2021). While the precise underlying mechanisms remain incompletely understood, olfactory deficits affect up to 90% of patients with Parkinson’s disease (PD) and approximately 85% of those with Alzheimer’s disease (AD). Importantly, these deficits often precede motor and cognitive symptoms by several years, reflecting early pathological changes within the olfactory bulb, olfactory tract, and associated neural networks.

This prolonged preclinical window provides a valuable opportunity for early detection. Standardized, accessible tools are now widely available for clinical screening; the two most prevalent are the University of Pennsylvania Smell Identification Test (UPSIT), which predominates in North America, and the Sniffin’ Sticks test, which is more widely used in Europe (Whitcroft and Hummel, 2019).

Growing evidence suggests that olfactory dysfunction may also serve as a clinical surrogate for early glymphatic system impairment. The glymphatic system functions as a cerebral waste-clearance pathway, facilitating the removal of metabolic byproducts and neurotoxic proteins through the convective movement of cerebrospinal fluid (CSF) within perivascular and interstitial spaces (Iliff et al., 2012). Because the nasal pathway serves as a primary route for CSF outflow, impaired glymphatic drainage may contribute to the localized accumulation of neurotoxic proteins within olfactory structures and downstream brain regions involved in odor processing.

Consequently, glymphatic dysfunction has emerged as a pivotal factor in the pathogenesis of numerous neuropathological disorders. Impaired clearance of β-amyloid and other metabolic waste products promotes protein aggregation, tau hyperphosphorylation, neuroinflammation, and progressive neuronal degeneration, ultimately driving cognitive decline and dementia (Ishida et al., 2022; Dagum et al., 2026). The deposition of these disease-specific proteins – including α-synuclein, hyperphosphorylated tau, and β-amyloid – within olfactory pathways, combined with the disruption of local neurotransmitter systems and neural circuitry, provides a plausible mechanistic link between olfactory deficits and subsequent cognitive impairment.

Consistent with this framework, Sanchez-Benavides et al. (Sanchez-Benavides et al., 2025) proposed that olfactory dysfunction may represent a primary clinical marker of glymphatic failure. Standardized olfactory testing therefore holds significant promise as a noninvasive, cost-effective screening tool to identify individuals at high risk for disease progression, offering valuable applications for both clinical trials and early therapeutic stratification (Dan et al., 2021; Gao et al., 2023).

Direct assessment of glymphatic function has historically relied on the intrathecal administration of magnetic resonance imaging (MRI) contrast agents, followed by serial imaging to track brain clearance (Ringstad et al., 2017; Klostranec et al., 2021). However, this approach is inherently invasive and carries risks associated with lumbar punctures and cumulative exposure to gadolinium-based contrast agents.

To circumvent these limitations, several noninvasive MRI techniques have been developed as surrogate measures of glymphatic kinetics. Perivascular Space (PVS) evaluation assesses the structural enlargement of perivascular spaces either visually or via automated image-analysis pipelines (Wardlaw et al., 2020; Shulyatnikova and Hayden, 2023; Menze et al., 2024; Waymont et al., 2024).

The Diffusion Tensor Image Analysis Along the Perivascular Space (DTI-ALPS) technique uses advanced imaging and yields a quantitative metric known as the ALPS index (Taoka et al., 2017, 2024; Menze et al., 2024; Zhang et al., 2025). The original developers reported that a higher ALPS index reflects greater radial diffusion of water molecules at the level of the lateral ventricular body, which is hypothesized to indicate more efficient glymphatic function.

While the ALPS index was designed to estimate water diffusivity along perivascular spaces where major white matter tracts run perpendicularly, both DTI-ALPS and PVS scoring face notable methodological criticisms.

DTI-ALPS does not directly measure glymphatic flow, CSF velocity, or solute clearance. Instead, it measures water diffusion characteristics presumed to correlate with glymphatic activity. The biological relationship between anisotropy and actual metabolic waste clearance remains incompletely validated.

The ALPS index can be heavily influenced by non-glymphatic factors, including age-related microstructural changes, white matter degeneration, demyelination, axonal injury, and small-vessel ischemic disease. Thus, a reduced index may reflect underlying tissue pathology rather than pure transport impairment. The index is calculated from a relatively small region near the lateral ventricles. Critics argue this limited sampling fails to accurately represent global, whole-brain glymphatic kinetics. ALPS indices are highly sensitive to MRI field strength, scanner manufacturer, diffusion acquisition parameters, region-of-interest placement, and post-processing algorithms, making cross-study comparisons difficult. Despite reported correlations between ALPS indices and cognitive impairment, sleep disturbances, and neurodegeneration, there is still no universally accepted gold standard proving that ALPS accurately quantifies human glymphatic function *in vivo* (Costa et al., 2024).

Similarly, utilizing visible PVS enlargement to infer glymphatic function is limited by the fact that it yields a structural, rather than functional, measurement. MRI visualization demonstrates static anatomy rather than dynamic fluid neuro-dynamics. While PVS enlargement implies altered fluid transport, it does not directly capture CSF movement, interstitial fluid exchange, or active waste clearance (Gouveia-Freitas and Bastos-Leite, 2021).

Despite these valid methodological constraints, both ALPS and PVS metrics remain the most accessible, noninvasive tools available for clinical glymphatic profiling, explaining their widespread utilization in current research.

To our knowledge, this is the first study to identify a distinct pathophysiological cluster of events characteristically associated with neuropathological diseases: olfactory dysfunction, glymphatic dysfunction, cognitive impairment, and sleep disturbances. The frequent, highly consistent co-occurrence of these abnormalities strongly suggests they share a common underlying physiological mechanism.

Accordingly, this article reviews current theories regarding the pathogenesis of olfactory dysfunction and this unique symptom cluster across a wide spectrum of neuropathological disorders. Special emphasis is placed on recent findings by the authors suggesting that nasal turbinate vasodilation and subsequent blood pooling may serve as a mechanical contributor to both olfactory and glymphatic dysfunction (Phillips et al., 2022).

## 2 Neuropathological disorders with impaired olfaction associated with a unique cluster of conditions: glymphatic dysfunction, cognitive impairment, and sleep disturbances

Many neuropathological disorders are associated with a cluster of conditions that include olfactory dysfunction, glymphatic system dysfunction, cognitive impairment, and sleep disturbances. In the following sections, specific findings associated with each neuropathological disorder will be described. This cluster of conditions is also outlined in Table 1 with references describing each condition cited.

**TABLE 1:** Citations documenting the unique cluster of symptoms associated with neuropathological disorders.

Neuropathological disorder	Olfactory dysfunction	Glymphatic dysfunction (Decreased MRI DTI-ALPS or increased perivascular spaces)	Cognitive impairment	Sleep disturbances
Parkinson’s Disease	Tolosa et al., 2021; Sui et al., 2019	Luo et al., 2024	Roheger et al., 2018	Iranzo et al., 2024
Alzheimer’s Disease	Roberts et al., 2016; McLaren and Kawaja, 2024; Liao et al., 2024	Keil et al., 2025	Irwin and Vitiello, 2019	Gottesman et al., 2024; Irwin and Vitiello, 2019
Frontotemporal Dementia	Tonacci and Billeci, 2018; Carnemolla et al., 2020	Jiang et al., 2023	Khalili et al., 2026	Bonakis et al., 2014
Traumatic Brain Injury	Liu X. et al., 2023	Iliff et al., 2014	Bryant et al., 2023	Ouellet et al., 2015
Idiopathic Intracranial Hypertension	Samanci et al., 2019	Schartz et al., 2024	Richey et al., 2024	Kabanovski et al., 2023
Schizophrenia	Ishizuka et al., 2010; Zurlo et al., 2025; Ouyang et al., 2024	Qi et al., 2026	McCutcheon et al., 2023	Ashton and Jagannath, 2020
Bipolar Disorder	Marin et al., 2023; Kazour et al., 2017	Chen et al., 2025	Sole et al., 2017	Marchetti et al., 2025
Depression	Khil et al., 2016; Sabiniewicz et al., 2022	Zhang F. et al., 2026	Rock et al., 2014	Rosenblum et al., 2025
Autism Spectrum Disorder *Neurodevelopmental Disorder	Bennetto et al., 2007; Crow et al., 2020; Rozenkrantz et al., 2015; Yang et al., 2022	Frigerio et al., 2025; Li et al., 2022	Velikonja et al., 2019	Reynolds and Malow, 2011

### 2.1 Parkinson’s disease

- *Olfactory dysfunction* affects approximately 90% of patients with PD and is recognized as a potential preclinical marker of motor symptoms (Tolosa et al., 2021). Longitudinal studies have demonstrated that hyposmia can precede motor manifestations by years or even decades. A meta-analysis of seven prospective studies confirmed that hyposmia is associated with a 3.84-fold increased risk of developing PD (95% CI 2.12–6.95) (Sui et al., 2019).
- *Glymphatic system dysfunction* in PD impairs the clearance of neurotoxic proteins like α-synuclein, contributing to neuropathological disease progression. MRI studies show evidence of reduced glymphatic flow such as enlarged perivascular spaces and a low ALPS index. Enlarged perivascular spaces in PD correlate with greater motor severity and cognitive decline (Lin et al., 2022; Luo et al., 2024).
- *Cognitive impairment* in PD is common, ranging from mild dysfunction to dementia, affecting memory, attention, and planning (Roheger et al., 2018). Up to 80% of patients may develop dementia after 10+ years. Symptoms include slow processing, word-finding issues, and visuospatial difficulties, often requiring lifestyle adjustments, therapy, and, when needed, medication.
- Parkinson’s disease is frequently associated with *sleep disturbances* including insomnia, fragmented sleep, rapid eye movement (REM), sleep behavior disorder (acting out dreams), and daytime sleepiness (Iranzo et al., 2024). These issues stem from brain changes, medication side effects, or symptoms like pain, anxiety, and the need to urinate frequently. Effective management involves tailored medications and behavioral changes.

### 2.2 Alzheimer’s disease

- *Olfactory dysfunction* is similarly prevalent in AD, affecting approximately 85% of patients in the early stages of disease (McLaren and Kawaja, 2024). Olfactory identification deficits precede detectable cognitive decline, including mild cognitive impairment (MCI) and even subjective cognitive decline (SCD), by several years (Liao et al., 2024). In the Mayo Clinic Study of Aging, Roberts and colleagues followed 1,430 cognitively normal participants and observed a dose-response relationship between worsening olfactory identification scores and incident amnestic MCI. Compared to the highest quartile of olfactory function, the lowest quartile demonstrated a hazard ratio of 2.18 (95% CI: 1.36–3.51) for developing amnestic MCI (Roberts et al., 2016). Furthermore, olfactory dysfunction predicted progression from amnestic MCI to AD dementia, with the lowest olfactory quartile showing a hazard ratio of 5.20 (95% CI: 1.90–14.20) compared to the highest quartile (Roberts et al., 2016). Recent investigations have linked olfactory dysfunction to cerebral β-amyloid deposition (Tian et al., 2022) and plasma biomarkers of neuropathological disease, including phosphorylated tau-181, glial fibrillary acidic protein, and neurofilament light chains (Mielke et al., 2025).
- *Glymphatic system dysfunction* is increasingly recognized as a key contributor to AD pathogenesis, primarily through impaired clearance of β-amyloid and tau proteins from the brain parenchyma (Ding et al., 2025; Mehrabadi and Samarghandian, 2026). Studies in rodent models have demonstrated an important role for the glymphatic system in the removal of β-amyloid and tau in the rodent brain and support the idea that its dysfunction contributes to the development of AD (Keil et al., 2025). Impaired glymphatic clearance may lead to β-amyloid accumulation, followed by tau phosphorylation and neural degeneration, which eventually causes cognitive impairment and dementia (Nedergaard and Goldman, 2020; Ding et al., 2025).
- *Alzheimer’s cognitive impairment* involves a progressive decline in memory, thinking, and reasoning skills, often starting with short-term memory loss and advancing toward difficulties with daily tasks, language, and behavior. While mild cognitive impairment can exist as a precursor with noticeable but less severe limitations, AD will usually progress, worsening functional decline (Irwin and Vitiello, 2019).
- *Sleep disturbances* in AD are common, appear early, and are characterized by fragmented sleep, nocturnal wandering, daytime sleepiness, and “sundowning” (increased agitation in late afternoon). These disruptions, resulting from neurodegeneration, often include reduced deep sleep (slow-wave sleep). Treatments emphasize behavioral strategies like consistent routines and bright light exposure (Gottesman et al., 2024).

### 2.3 Frontotemporal dementia

- *Olfactory dysfunction* is highly prevalent in frontotemporal dementia (FTD), affecting up to 96% of cases. It is characterized by impaired odor identification rather than just sensory perception, likely due to atrophy in temporal and orbitofrontal brain regions, making it a potential biomarker for diagnosis and disease progression (Tonacci and Billeci, 2018; Carnemolla et al., 2020).
- Frontotemporal dementia is increasingly linked to *glymphatic system dysfunction* as a failure in the brain’s perivascular waste clearance mechanism that likely accelerates the buildup of toxic proteins like tau and TDP-43. Studies show reduced glymphatic clearance, particularly in the forebrain and midbrain, correlating with neurodegeneration and symptom worsening in behavioral variant FTD (bvFTD) (Jiang et al., 2023).
- *Cognitive impairment* in FTD primarily involves progressive deficits in executive function, planning, and language, often with relatively spared memory and visuospatial skills early in the disease course. Caused by atrophy in the frontal and temporal lobes, symptoms include poor decision-making, mental rigidity, severe language impairment, and impaired social cognition (loss of empathy) (Khalili et al., 2026).
- *Sleep disturbances* are common and often early features of FTD, affecting 33%–76% of patients. Sleep disturbances may be equal to or more severe than those seen in Alzheimer’s disease and may occur earlier in the disease course. Key issues include insomnia, excessive daytime sleepiness, nocturnal awakenings, and disrupted circadian rhythms. These disturbances, driven by neurodegeneration in brain regions regulating sleep, increase caregiver burden and may accelerate cognitive decline (Bonakis et al., 2014).

## 3 Other neuropathological disorders associated with the same cluster of symptoms

### 3.1 Traumatic brain injury

- Approximately 20% to 68% of patients with traumatic brain injury experience some degree of olfactory dysfunction. The exact percentage of smell loss or reduction varies depending on the severity of the head trauma and the diagnostic methods used to evaluate the patient’s sense of smell (Liu X. et al., 2023). Head trauma commonly causes *olfactory dysfunction* due to the vulnerable anatomic location of olfactory nerve fibers extending from the olfactory bulb near the cribriform plate (Kazour et al., 2017; Liu X. et al., 2023).
- Traumatic brain injury (TBI) causes *significant glymphatic dysfunction*. In animal models of TBI, the cerebrospinal fluid – interstitial fluid (CSF–ISF) exchange is reduced by approximately 60% (Iliff et al., 2014). This disruption leads to the accumulation of waste products like β-amyloid and p-tau, exacerbating neuroinflammation, and long-term neurodegeneration, such as chronic traumatic encephalopathy (Peters and Lyketsos, 2023).
- Traumatic brain injury causes *cognitive impairment* through damaged neural pathways, leading to deficits in attention, memory, executive function, and information processing speed. These impairments, which can range from mild to severe, often disrupt daily life and necessitate rehabilitation, including cognitive training and sometimes medication to manage symptoms like poor focus or memory loss (Bryant et al., 2023).
- *Sleep disturbances* are highly prevalent following TBI, affecting 30–70% of individuals. Common issues include insomnia, fatigue, and hypersomnia. TBI-related sleep disturbances can stem from neurochemical changes, pain, depression, and disruptions to the circadian rhythm, often requiring a combination of behavioral therapies, such as Cognitive Behavioral Therapy for Insomnia (CBT-I), and careful pharmacological management (Ouellet et al., 2015).

### 3.2 Idiopathic intracranial hypertension

- Intracranial hypertension, particularly idiopathic intracranial hypertension (IIH) is associated with significant *olfactory dysfunction*, primarily affecting the odor detection threshold more than odor identification. Up to 57% of IIH patients meet criteria for hyposmia on standardized testing. This impairment appears to be reversible with reduction of intracranial pressure (Samanci et al., 2019).
- *Glymphatic dysfunction* is a suspected key factor in the pathophysiology of IIH, representing a potential failure of the brain’s waste-clearance system (Schartz et al., 2024). Impaired clearance of interstitial fluid (ISF) leads to increased intracranial pressure (ICP), with recent imaging studies showing that changes in glymphatic transit and perivascular spaces may potentially be used as biomarkers to support the diagnosis of IIH (Schartz et al., 2024).
- Idiopathic intracranial hypertension frequently causes multidomain *cognitive impairment*, notably affecting attention, working memory, processing speed, and executive function. These impairments, often described as “brain fog,” are associated with elevated intracranial pressure, chronic headache, and low quality of life. Cognitive issues can persist even after treatment but may improve with pressure reduction (Richey et al., 2024).
- Idiopathic intracranial hypertension is frequently associated with significant *sleep disturbances*, particularly obstructive sleep apnea (OSA), which affects up to 64% of patients (Kok et al., 2023). The association is particularly notable in men with IIH, where OSA was found in 9 of 11 (82%) in one prospective study, though BMI was the strongest predictor of OSA diagnosis rather than IIH itself (Kabanovski et al., 2023). These patients experience poor sleep quality, increased daytime sleepiness, and nocturnal disturbances. OSA can exacerbate IIH symptoms, including headaches and papilledema, making sleep assessments vital (Kabanovski et al., 2023).

In addition to neuropathological disease, this same unique cluster of symptoms is present in many psychiatric conditions.

## 4 Psychiatric conditions

### 4.1 Schizophrenia

- Up to 80% of patients with schizophrenia experience some form of *olfactory dysfunction* (Ishizuka et al., 2010), particularly in higher-order tasks like odor identification and discrimination, with effect sizes typically around one standard deviation compared to healthy controls (Zurlo et al., 2025). These impairments appear early in the illness course and may serve as a marker of disease progression. Poorer olfactory performance is associated with greater negative symptoms, longer illness duration, lower anticipatory pleasure, and worse cognitive function (Ouyang et al., 2024).
- Schizophrenia is linked to *glymphatic system dysfunction* that likely contributes to cognitive decline and the pathophysiology of the disease. Studies show reduced DTI-ALPS indices in patients, indicating lower fluid transport efficiency in the brain, with greater impairments correlated to more severe cognitive deficits (Qi et al., 2026). This dysfunction may involve altered AQP4 expression (the most abundant water channel in the CNS) and neuroinflammation (Wu et al., 2020; Peng et al., 2023).
- *Cognitive impairment* is a core feature of schizophrenia, affecting nearly 98% of patients. These impairments – including deficits in working memory, attention, processing speed, and executive function – are often present before the onset of psychosis and predict long-term functional impairment. These cognitive impairments are relatively stable, rarely improve with antipsychotic medication, and often predict functional outcomes more strongly than psychotic symptoms (McCutcheon et al., 2023).
- Schizophrenia commonly causes severe sleep disturbances, including insomnia, circadian rhythm reversals (awake at night, sleepy by day), and fragmented sleep, affecting up to 80% of patients. These issues worsen psychotic symptoms and cognitive performance. Management typically involves sedating antipsychotics, melatonin, and cognitive behavioral therapy (CBT) (Ashton and Jagannath, 2020).

### 4.2 Bipolar disorder

- Patients with bipolar disorder (manic depression) demonstrate *olfactory dysfunction* that persist across mood states, though the severity varies (Marin et al., 2023). Studies utilizing standardized smell tests often show that between 50% and 80% of bipolar patients exhibit impairments, specifically in odor identification and olfactory sensitivity, compared to healthy individuals (Kazour et al., 2017). During acute manic episodes, patients show particularly pronounced impairment in identifying positive odors and rate these smells as less pleasant compared to euthymic patients and healthy controls (Kazour et al., 2022).
- *Glymphatic system dysfunction* is associated with cognitive impairment, neuroinflammation, and prolonged illness duration in bipolar disorder. Research indicates that reduced glymphatic function, often measured by DTI-ALPS MRI, is linked to sleep disruption and increased β-amyloid accumulation in bipolar disorder patients (Chen et al., 2025).
- Bipolar disorder causes *persistent cognitive impairment* – most notably in attention, memory, and executive function – that often continues even during periods of mood stability (euthymia). Affecting functional, everyday life, these deficits are associated with structural brain changes, such as reduced cortical thickness in prefrontal areas, and are increased by the number of past mood episodes (Sole et al., 2017).
- Bipolar disorder and *sleep disturbances* are deeply intertwined, with sleep disturbances serving as both a symptom and a trigger for mania or depression. Nearly 69%–99% of individuals experience reduced sleep during mania, while 38%–78% experience excessive sleep (hypersomnia) during depression. Establishing a strict, consistent sleep schedule, managing stress, and adjusting medications with a doctor are critical management strategies (Marchetti et al., 2025).

### 4.3 Depression

- Research indicates that approximately 20% to 50% of patients with depression experience some degree of *olfactory* dysfunction (Sabiniewicz et al., 2022). Olfactory dysfunction and depression are bidirectionally related, with olfactory dysfunction both occurring in patients with major depressive disorder (MDD) and predicting the subsequent development of depression in older adults. Recurrent depressive episodes and longer disease duration are associated with greater olfactory dysfunction. Patients with frequent depression-related hospitalizations demonstrate elevated odds of odor identification impairment compared to non-depressed controls (Khil et al., 2016).
- *Glymphatic system dysfunction* is increasingly recognized as a key contributor to major depressive disorders. A primary meta-analysis of 11 studies (comprising 1470 patients and 962 healthy controls) revealed a significant reduction in the DTI-ALPS index in MDD patients consistent with glymphatic dysfunction (Zhang F. et al., 2026).
- *Cognitive impairment* is now recognized as a core feature of major depressive disorder, not merely an epiphenomenon of low mood. Meta-analytic data demonstrate moderate deficits across memory, attention, and processing speed (Cohen’s d ranging from −0.34 to −0.65) during acute episodes (Rock et al., 2014).
- *Sleep disturbances* are both a core symptom and a risk factor for depression. Sleep alterations in MDD include the presence of insomnia or hypersomnia and aberrations in sleep macro- and micro-structure, including prolonged duration of the first rapid eye movement (REM) episode, decreased slow-wave sleep (SWS), disturbed sleep continuity, and decreased steepening of aperiodic neural activity (Rosenblum et al., 2025).

## 5 Neurodevelopmental disorder

### Autism spectrum disorder

Autism Spectrum Disorder (ASD) is primarily classified as a neurodevelopmental disease. However, from a biological standpoint, it is characterized by specific neuropathology involving atypical brain development, abnormal cortical organization, and altered synaptic connectivity. Pathology varies among individuals with ASD, and research reveals consistent neurological differences.

Individuals with ASD also experience the same unique cluster of symptoms including olfactory dysfunction, glymphatic dysfunction, cognitive impairment, and sleep disturbances.

- Over 50% of individuals with ASD experience unusual smell sensitivities or *olfactory dysfunction.* Rather than a uniform loss of smell, the dysfunction typically manifests as atypical sensory processing (hyper- or hypo-reactivity) and discrimination (Yang et al., 2022). These individuals also demonstrate olfactory dysfunction in detection thresholds, though the pattern of deficits shows substantial heterogeneity across studies (Bennetto et al., 2007; Crow et al., 2020). A particularly striking finding is the absent or profoundly altered sniff response in children with ASD (Rozenkrantz et al., 2015).
- *Glymphatic system dysfunction* is an emerging pathophysiologic factor in the etiology of ASD, marked by impaired brain metabolic waste clearance, especially during sleep. Studies have found that ASD individuals have an increased burden of perivascular spaces, and that the increased burden of perivascular spaces correlates with ASD severity (Frigerio et al., 2025). Studies show reduced DTI-ALPS indices in ASD, indicating poor perivascular flow and potential neuroinflammation. This dysfunction likely contributes to neurodevelopmental abnormalities and symptom severity (Li et al., 2022).
- *Cognitive impairment* in ASD involves key deficits in social cognition, executive functioning, and sensory processing. Key challenges include reduced cognitive flexibility, impaired planning, and slower processing speeds. These deficits, often linked to structural and neuroinflammatory factors, can lead to increased risks of cognitive decline with age (Velikonja et al., 2019).
- Up to 80% of individuals with ASD experience *sleep disturbances*, including insomnia, frequent night waking, and short sleep duration, often due to irregular melatonin levels, anxiety, or sensory sensitivities. Poor sleep can worsen core ASD behaviors, such as hyperactivity, anxiety, and learning difficulties. Effective interventions include consistent bedtime routines, behavioral approaches, managing the sleep environment, and sometimes melatonin (Reynolds and Malow, 2011).

## 6 Alternative mechanisms proposed to be involved in the development of neuropathological disease

There are five core mechanisms that are frequently cited in the literature as being associated with/causing neuropathological disease. These mechanisms are neuroinflammation, vascular dysfunction, mitochondrial dysfunction, blood-brain barrier (BBB) disruption, and protein aggregation. All of these mechanisms are deeply interconnected in a self-perpetuating, pathogenic cycle causing neuropathological disease. These mechanisms form a system-level network where the breakdown of one pathway rapidly accelerates the failure of others (Hein et al., 2025).

- Neuroinflammation is triggered by resident immune cells (microglia and astrocytes) detecting abnormal proteins or cellular debris. These inflammatory mediators directly cause synaptic damage, trigger neuronal apoptosis, and promote further BBB permeability (Zhang et al., 2023).
- Vascular dysfunction is characterized by impaired cerebral blood flow, endothelial cell injury, and the loss of critical support cells like pericytes within the neurovascular unit (Raz et al., 2016).
- Mitochondria are highly sensitive to oxidative stress and play a critical role in cellular energy (ATP) production, calcium buffering, and apoptosis (Hein et al., 2025).
- The blood-brain barrier is maintained by tight junctions between brain microvascular endothelial cells. Systemic inflammation works to loosen these tight junctions, severely compromising BBB integrity (Sarhan et al., 2025).
- The misfolding and accumulation of pathological proteins occurs in neuropathological disorders. These abnormal aggregates are highly toxic to surrounding cells (Sarhan et al., 2025).

Although the above mechanisms are proposed to be involved in the development of a wide variety of neuropathological disorders, the initial events that trigger the development of any one of these processes is not well understood.

## 7 Known pathophysiological mechanisms of olfactory dysfunction

Specific mechanisms have been associated with olfactory dysfunction. The olfactory bulb (OB), entorhinal cortex (EC), and transentorhinal cortex are among the first cortical sites to accumulate neurofibrillary tangles (NFTs) in AD. Known pathological involvement of the olfactory regions include: (1) aggregation of misfolded proteins in olfactory structures, including hyperphosphorylated tau, β-amyloid and α-synuclein (2) neurotransmitter disruption, and (3) neural network alterations.

### 7.1 Protein aggregation and neuropathology

The neuropathological substrate of olfactory dysfunction in neuropathological diseases involves the deposition of disease-specific misfolded proteins in olfactory structures. In a large autopsy cohort of 536 individuals, the severity of olfactory bulb hyperphosphorylated tau, β-amyloid, and α-synuclein pathology correlated significantly with increasing Braak stages, a system used to classify the degree of pathology in PD and AD (Attems et al., 2014).

- Histopathological examination of post-mortem olfactory bulbs from AD patients reveals severe structural alterations in olfactory glomeruli, including significant β-amyloid accumulation, morphologic damage, altered neurotransmitter levels, and microgliosis (Son et al., 2022).
- Ultrastructural analysis demonstrates distorted postsynaptic densities and decreased presynaptic vesicles, indicating synaptic dysfunction (Son et al., 2022). These pathological changes induce a cascade of molecular processes including oxidative damage, neuroinflammation, and cytosolic disruption leading to neuronal death (Attems et al., 2014).

### 7.2 Neurotransmitter system disruption

Damage to cholinergic, serotonergic, and noradrenergic systems contributes to olfactory dysfunction, with such damage being most pronounced in diseases characterized by severe anosmia (Attems et al., 2014). Recent mechanistic studies have identified early locus coeruleus noradrenergic axon loss as a driver of olfactory dysfunction in AD (Meyer et al., 2025). In AD mouse models, distinct loss of noradrenergic input to the olfactory bulb coincides with impaired olfaction before β-amyloid plaque appearance, mediated by microglial phagocytosis of locus coeruleus axons. This finding has been confirmed in post-mortem olfactory bulbs from patients with early AD (Meyer et al., 2025).

Interestingly, stereological studies have demonstrated an increased number of dopaminergic periglomerular neurons in the olfactory bulbs of AD, PD, and frontotemporal dementia patients compared to controls (Mundinano et al., 2011). The increased number of neurons may represent a compensatory mechanism in response to early degeneration of other neurotransmitter systems, potentially contributing to the observed olfactory dysfunction (Mundinano et al., 2011).

### 7.3 Neural network alterations

Olfactory dysfunction reflects issues across multiple levels of the olfactory pathway, including the olfactory epithelium, olfactory bulb and tract, primary olfactory cortices, and secondary targets (Attems et al., 2014). Functional neuroimaging studies reveal abnormal connectivity between gray matter regions (dorsolateral prefrontal cortex, anterior entorhinal cortex, fronto-orbital cortices) and white matter tracts (posterior and superior corona radiata) in patients with PD-associated hyposmia (Wang et al., 2022).

Age-related atrophic changes in integral olfactory components – including the olfactory tubercle in the piriform cortex, lateral entorhinal cortex, and para-amygdaloid complex – contribute to olfactory dysfunction (Yeo et al., 2024). These structures project to the orbitofrontal cortex, insular cortex, and dorsal hippocampus, which also experience atrophy in neurodegenerative disorders (Yeo et al., 2024). Aberrant neural oscillations and synaptic plasticity deficits in the olfactory bulb and piriform cortex further compromise olfactory neural networks in preclinical AD (Yan et al., 2022).

## 8 Proposed pathophysiologic conditions leading to olfactory dysfunction

The traditional thinking about olfactory dysfunction in neuropathological disease suggests hyperphosphorylated tau appears in the locus coeruleus and transentorhinal/entorhinal regions at the earliest stages (Braak I–II), well before neocortical involvement (Kaufman et al., 2018). However, there is very little understanding of why these olfactory brain regions would be affected first before other brain regions. There have been many theories as to why the olfactory region is uniquely susceptible to this initial damage. The following are the most widely accepted theories.

### 8.1 The vector hypothesis

The vector hypothesis provides a plausible biological explanation for the vulnerability of the olfactory system in neurodegeneration (Yeo et al., 2024). The olfactory cranial nerve directly communicates with the external environment via the cribriform plate, rendering it susceptible to external pathogenic stressors. Pathogens may penetrate through the olfactory epithelium and olfactory bulb, causing progressive brain damage over time (Yeo et al., 2024). It has been hypothesized that viral infections can enter through the nose, spread to the olfactory bulb, and then move along the brain neuron pathway to the entorhinal cortex before spreading to other regions (van Riel et al., 2015). This anatomical vulnerability, combined with the olfactory system’s extensive neural connections, may facilitate the spread of pathological proteins throughout the brain.

### 8.2 Complementary mechanisms make the olfactory region vulnerable

Several complementary mechanisms have been invoked to explain the regional predilection of olfactory regions, including the hypothesized convergence of region-specific molecular profiles, cell-type vulnerability, high neuronal activity, connectivity-driven prion-like seeding, and impaired mitochondrial clearance. The cell type vulnerability includes the fact that the entorhinal cortex has the highest regional expression of aggregation-prone 4R tau (a specific isoform of the tau protein) and tau kinases, while the cerebellum – which is spared from tau pathology – has the lowest (Hu et al., 2017).

## 9 Olfactory dysfunction in neuropathological disorders is potentially due to nasal turbinate vasodilation.

The authors propose an alternate explanation for the olfactory region being the first area affected in neuropathological conditions.

**FIGURE 1:**
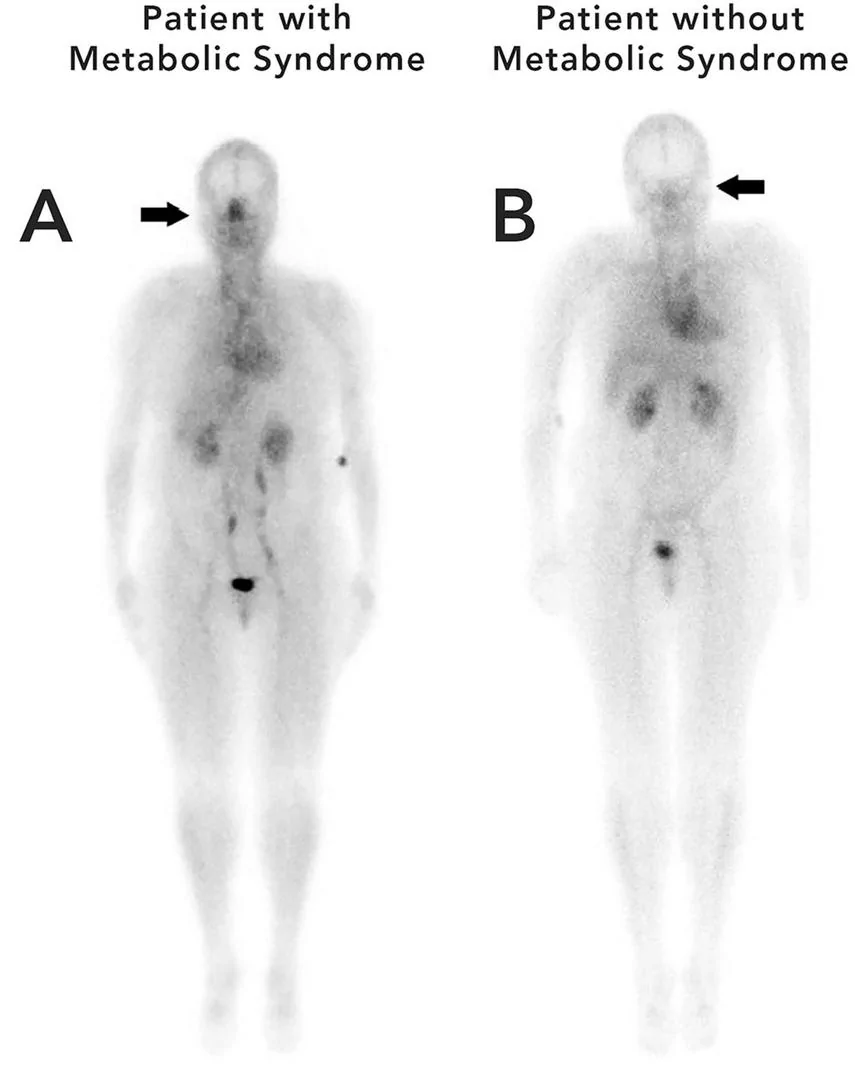
An example of whole-body blood pool scans of two patients with similar BMIs. One figure **(A)**, with signs and symptoms of metabolic syndrome, shows increased blood pooling in the nasal area (arrow). **(B)** A patient without any signs or symptoms of metabolic syndrome with the exception of an elevated BMI, shows no blood pooling in the nasal area (arrow). Reproduced Figure from Phillips, Sheesley, and Schwartz, *Front Neurosci*, 2026, Jan 20.

In a prior study, the authors reported that significantly increased nasal blood pooling (Figure 1) occurs in patients with the metabolic syndrome conditions of hypertension, diabetes, and obesity (Phillips et al., 2022). This increased nasal blood pooling was considered to be due to increased activation of the parasympathetic system causing nasal turbinate vasodilation. It is proposed by the authors that metabolic syndrome conditions induce nasal turbinate vasodilation and blood pooling which leads to meningeal *lymphatic obstruction* and subsequent *glymphatic obstruction*, thereby impairing the brain’s waste clearance system. This glymphatic obstruction accelerates the accumulation of neurotoxic proteins (e.g., β-amyloid), fostering cognitive impairment, and increasing dementia risk.

Consistent with this hypothesis is that metabolic syndrome conditions are generally associated with *olfactory dysfunction*, resulting in reduced smell sensitivity and identification, particularly in individuals with obesity, diabetes, or hypertension.

Glymphatic dysfunction due to nasal turbinate lymphatic obstruction would explain perivascular space dilatation and lower DTI-ALPS indices found on MRI in patients with olfactory dysfunction. There is significant experimental research showing CSF clearance eventually ends up in superficial and deep cervical lymphatics (Johnston et al., 2004). Many researchers believe that the majority of CSF cleared from the glymphatic system moves into meningeal lymphatics (Aspelund et al., 2015). Research in animal models has shown the majority of CSF in the meningeal lymphatics moves through the cribriform plate into larger lymphatics located in the nasal turbinates and then into superficial and deep cervical lymphatic vessels (Nagra et al., 2006; Chae et al., 2024; Algin et al., 2025). Although the precise pathways and importance of each of these larger lymphatic pathways remains controversial, particularly in humans, CSF lymphatic obstruction at the level of the nasal turbinates would lead to glymphatic dysfunction.

## 10 Metabolic syndrome as an initial event in neuropathological disorders with impaired olfaction – a proposal by the authors

Although metabolic syndrome has been known as an associated risk factor for many neuropathological diseases, the authors hypothesize its presence may have a more profound impact. An increased risk of metabolic syndrome and olfactory dysfunction is present in each of the previously described neuropathological disorders in Sections 2–5, and provides support for the authors’ hypothesis of nasal turbinate lymphatic obstruction as a common etiology. An example of nasal turbinate vasodilation and blood pooling in a patient with metabolic syndrome as compared to a patient without metabolic syndrome is shown in Figure 1.

**FIGURE 2:**
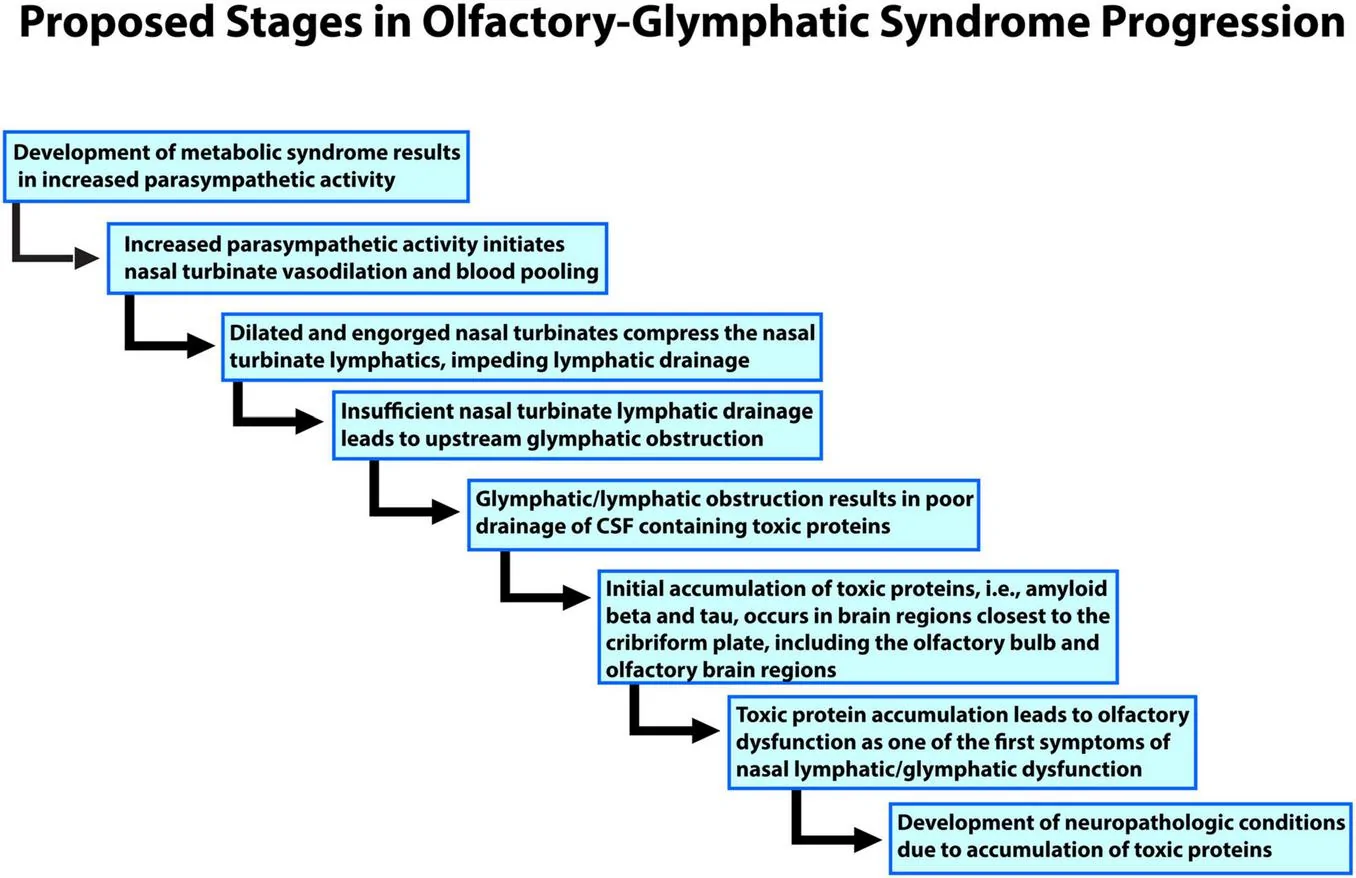
A speculative series of events proposed by the authors with metabolic syndrome initiating glucose dysregulation and increased parasympathetic activity, leading to eventual nasal turbinate lymphatic obstruction and glymphatic dysfunction, and the development of neuropathological conditions. Original flow diagram created by the authors.

Figure 2 represents the authors’ conceptual model of metabolic syndrome initiating a cascade of events leading to eventual glymphatic dysfunction and the development of neuropathological disorders in the Olfactory-Glymphatic Syndrome.

**FIGURE 3:**
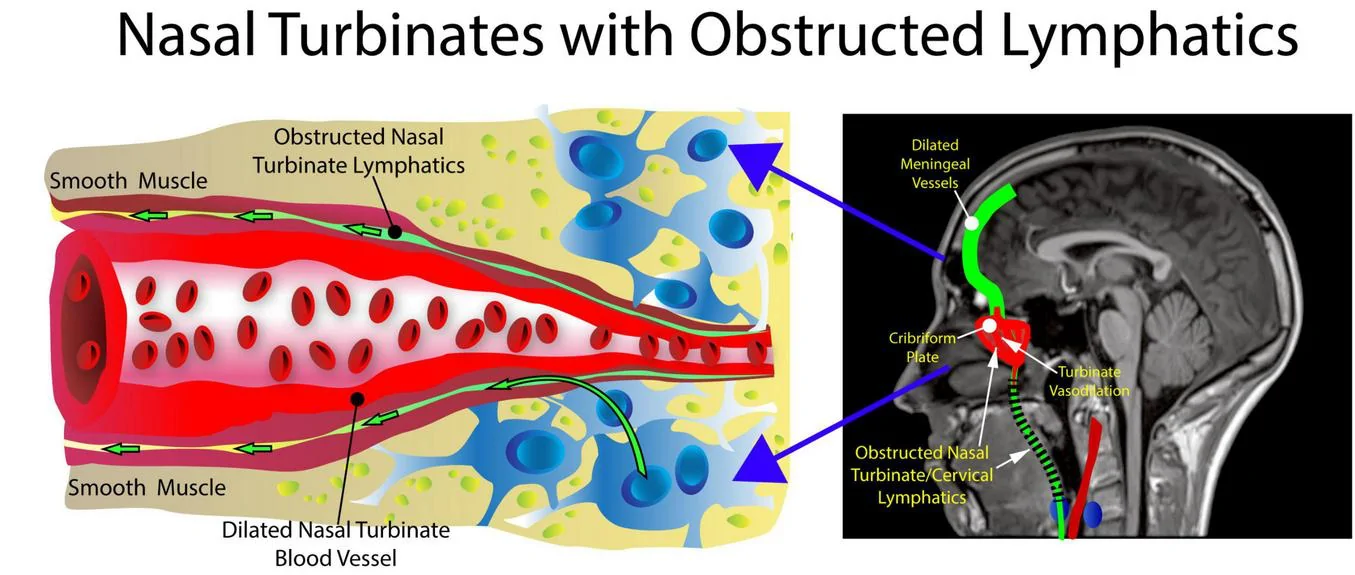
Conceptual illustration of abnormal anatomy within the nasal turbinates in patients with metabolic syndrome. Vasodilation and blood pooling occur in the area of the nasal turbinates due to increased parasympathetic activity. The expansion of the blood vessels, as proposed by the authors, would result in impingement/obstruction of the normal nasal lymphatic and glymphatic flow of CSF waste products containing toxic proteins. Reproduced Figure from Phillips and Schwartz, *Front Aging Neurosci*, 2024, Oct 21.

Figure 3 illustrates the authors’ theory of nasal turbinate vasodilation obstructing normal CSF flow through lymphatic vessels in the nasal turbinates in patients with metabolic syndrome.

## 11 Increased risk of metabolic syndrome in various neuropathological disorders

The previously described neuropathological disorders in Sections 2–5 are associated with an increased risk of metabolic syndrome. Greater detail describing this association is included in the following section.

### 11.1 Parkinson’s disease

*Metabolic syndrome* is associated with a roughly 24% to 40% increased risk of developing Parkinson’s disease (PD). This cluster of conditions, which is modifiable through lifestyle, may accelerate PD progression and severity, making management of metabolic health crucial for PD prevention and care (Zhong et al., 2024; Invernizzi et al., 2025).

### 11.2 Alzheimer’s disease

*Metabolic syndrome* significantly increases the risk of dementia and accelerates cognitive decline in Alzheimer’s disease (Machado-Fragua et al., 2022; Lee et al., 2025). Known as “type 3 diabetes,” brain insulin resistance impairing glucose metabolism, has been reported to foster β-amyloid plaque accumulation and neuroinflammation. Managing hyperglycemia and insulin resistance is considered a potential method to slow cognitive decline (Michailidis et al., 2022). When considering Alzheimer’s disease specifically, there is significant support for the association of single metabolic syndrome components, particularly hyperglycemia and diabetes (Zuin et al., 2021).

### 11.3 Frontotemporal dementia

Frontotemporal dementia is strongly linked to *metabolic syndrome*, characterized by glucose metabolism dysregulation, hyperinsulinemia, low HDL cholesterol, and high triglycerides. Patients with behavioral-variant FTD (bvFTD) frequently show eating changes, such as binge-eating and sugar craving, which may cause peripheral insulin resistance and weight changes that potentially affect disease progression (Ahmed et al., 2014).

### 11.4 Traumatic brain injury

Traumatic brain injury triggers systemic *metabolic syndrome*, often characterized by persistent glucose intolerance, dyslipidemia, hypertension, and weight gain, which worsens long-term neurological outcomes. This condition involves chronic neuroinflammation, mitochondrial dysfunction, and disrupted glucose metabolism – initially hypermetabolism followed by prolonged hypometabolism – that mirror neurodegenerative diseases (Izzy et al., 2022).

### 11.5 Idiopathic intracranial hypertension

Idiopathic intracranial hypertension is increasingly recognized as a metabolic disease rather than just a neuro-ophthalmic disorder, characterized by obesity, insulin resistance, and systemic metabolic dysfunction beyond what is driven by obesity alone. Key features include *metabolic syndrome*, polycystic ovary syndrome (PCOS), and androgen excess, requiring holistic, multidisciplinary care (Yiangou et al., 2023).

### 11.6 Schizophrenia

*Metabolic syndrome* is highly prevalent in individuals with schizophrenia, with rates roughly 2-3 times higher than the general population, affecting over one-third of patients. This combination of obesity, hypertension, high triglycerides, and glucose intolerance significantly reduces life expectancy. Key drivers include second-generation antipsychotic side effects, sedentary lifestyles, and poor diet (Goldfarb et al., 2022).

### 11.7 Bipolar disorder

Individuals with bipolar disorder (BD) have a significantly higher prevalence of metabolic syndrome, 32.7% – often 1.5 to 3 times higher than the general population – leading to elevated cardiovascular risk, type 2 diabetes, and premature mortality. This co-occurrence is driven by medication side effects (weight gain, insulin resistance), sedentary lifestyles, chronic inflammation, and genetic factors (Salari et al., 2025).

### 11.8 Depression

Depression and *metabolic syndrome* are bidirectionally associated, with a pooled relative risk of approximately 1.29–1.52 depending on the direction and study design. The relationship is mediated in part by chronic inflammation and oxidative stress and has important implications for antidepressant selection and integrated management (Meng et al., 2025).

### 11.9 Autism spectrum disorder

Individuals with ASD have a significantly higher risk of *metabolic syndrome* – including obesity, hypertension, dyslipidemia, and type 2 diabetes – compared to the general population. This increased risk is linked to genetic factors, unhealthy dietary habits, physical inactivity, and the side effects of psychotropic medications (Chieh et al., 2021).

## 12 Clinical applications and diagnostic utility

### 12.1 Olfactory testing methodologies

As previously described, standardized testing can be used to screen patients for olfactory dysfunction by the evaluation of olfactory thresholds, odor identification and discrimination, and olfactory memory using the University of Pennsylvania Smell Identification Test (UPSIT) (Doty et al., 1984; Liu S. et al., 2023), the Brief Smell Identification Test (B-SIT) (Cao et al., 2022), and Sniffin’ Sticks tests (Roberts et al., 2016). These assessments are simple, non-invasive, and cost-effective, making them attractive for large-scale screening applications.

Olfactory functional magnetic resonance imaging (fMRI) offers a relatively objective assessment of olfactory capabilities and shows promise for exploring neural mechanisms of olfactory damage in neuropathological disorders (Han et al., 2019). This neuroimaging approach may complement behavioral testing in research and clinical settings.

### 12.2 Diagnostic performance

Meta-analytic evidence demonstrates that olfactory function testing exhibits substantial diagnostic efficacy for AD and MCI. For AD diagnosis, the combined sensitivity is 0.79 (95% CI: 0.71–0.85), specificity is 0.78 (95% CI: 0.69–0.84), and area under the curve is 0.85 (95% CI: 0.82–0.88) (Liu et al., 2025). For MCI, sensitivity is 0.67 (95% CI: 0.54–0.78), specificity is 0.79 (95% CI: 0.71–0.86), and area under the curve is 0.81 (95% CI: 0.77–0.84) (Liu et al., 2025).

Importantly, subjective olfactory assessments correlate weakly with objective smell test performance, with only fair agreement (Markovic-Obiago et al., 2025). Subjective smell ratings do not correlate with motor function, whereas objective smell test results do, emphasizing the necessity of objective olfactory testing in research and clinical practice (Markovic-Obiago et al., 2025).

### 12.3 Risk stratification and screening

Olfactory testing demonstrates utility for risk stratification in prodromal populations (De Cleene et al., 2024; Markovic-Obiago et al., 2025). The correlation between olfactory dysfunction and other prodromal features – including REM sleep behavior disorder and bradykinesia – supports its integration into comprehensive risk assessment protocols (Markovic-Obiago et al., 2025). In cognitively normal individuals, olfactory dysfunction identifies those at increased risk for progression to MCI and dementia, enabling targeted surveillance and early intervention (Roberts et al., 2016).

The association between olfactory dysfunction and plasma biomarkers of AD and neurodegeneration suggests that combined assessment strategies may enhance diagnostic accuracy (Shrestha et al., 2025). Olfactory testing may prove particularly valuable in clinical trials for identifying subjects in early, preclinical stages of neurodegeneration and stratifying cohorts according to underlying neuropathology (Attems et al., 2014).

### 12.4 Limitations and future directions

Despite promising evidence, several limitations warrant consideration. Olfactory dysfunction is not specific to neuropathological disorders and can result from normal aging, sinonasal disease, head trauma, and other conditions (Dan et al., 2021). Establishing disease-specific patterns of olfactory dysfunction and integrating olfactory testing with other biomarkers will be essential for improving diagnostic specificity.

The strength of the authors’ hypothesis regarding a unique cluster of symptoms associated with neuropathological diseases and a possible common etiology results from their own research comparing radiologic whole-blood volume scans in patients with and without metabolic disease, and from an extensive search of the literature. Although a wide range of neuropathological diseases have been discussed, including neurodegenerative disorders, traumatic brain injuries, psychiatric diseases, and neurodevelopmental disease, the commonality of an elevated risk of metabolic syndrome and a unique cluster of symptoms is frequently associated with each of the entities. The authors have designated these findings as the “olfactory-glymphatic syndrome.”

The authors acknowledge the methodological and conceptual limitations of their article and realize future studies involving stronger justification and further validation leading to lymphatic/glymphatic obstruction and eventual cognitive impairment is warranted.

## 13 Future strategies for validation of the olfactory-glymphatic syndrome

The authors believe the best strategy for validation of the olfactory-glymphatic syndrome and confirmation of the unique cluster of symptoms described in this article would be to focus on the nasal turbinates in each of the neuropathologic conditions described. The topic of nasal turbinates in relationship to neuropathologic disorders is rarely mentioned in the current literature, even though the nasal turbinates have been clearly recognized as significant transporters of CSF waste via the nasal turbinate lymphatic vessels (Johnston et al., 2004; De Leon et al., 2017; Sun et al., 2018; Zhou et al., 2022; Chae et al., 2024; Algin et al., 2025).

In prior studies, the authors have shown significant correlations of nasal turbinate blood pooling in patients with diabetes, hypertension, elevated BMI, and sleep apnea (Phillips et al., 2022). Further studies comparing patients versus controls could be conducted utilizing scintigraphic nasal blood pool imaging to assess nasal turbinate blood pooling in each of the conditions described in this article.

Other imaging studies of the nasal turbinates could be performed to assess nasal turbinate enlargement in patients with these clustered conditions utilizing MRI or CT as compared to matched controls (Peporte et al., 2026). A retrospective study could easily be conducted with widely available MRI or CT scans.

Studies involving turbinate volume assessments such as those reported by Rodrigues et al., who described turbinate hypertrophy, could be performed. Rodrigues et al. were able to calculate nasal airway volume in obese patients using CT and the analysis of transaxial images (Rodrigues et al., 2020). Patients with sleep apnea exhibited significantly increased nasal turbinate hypertrophy.

It would also be possible to compare patients versus controls regarding nasal turbinate hypertrophy using acoustic rhinometry. This technology uses transmitted sound waves to measure the cross-sectional area of the nasal airway, typically 2–6 cm from the anterior nasal opening. Acoustic rhinometry measurements were shown to correlate well with the size of the inferior turbinates (Lenders and Pirsig, 1990). Acoustic rhinometry could also be performed rapidly as a screening tool for a selection of cases to be included in a more detailed study.

Longitudinal studies could also be performed linking nasal turbinate hypertrophy and vasodilation to lymphatic/glymphatic obstruction and eventual cognitive impairment. The precise biological mechanisms linking olfactory dysfunction to neuropathological disorders remain incompletely understood, necessitating further investigation of molecular pathways, genetic variants, and environmental factors (De Cleene et al., 2024; McLaren and Kawaja, 2024). Longitudinal studies examining temporal relationships between olfactory changes and biomarker evolution will provide critical insights into disease progression (Shrestha et al., 2025).

## 14 Therapeutic possibilities aimed at olfactory and glymphatic dysfunction

Early diagnosis of olfactory dysfunction as a risk factor of future development of neuropathological disorders would allow for much earlier intervention with therapies. The understanding of the clustering of findings associated with olfactory dysfunction could provide novel early therapeutic avenues aimed at any of these clustered disorders. Interventions directly targeting olfactory dysfunction have the potential to modify disease trajectories which represent an important avenue for therapeutic development. Zhang et al. have recently reviewed a wide variety of methodologies to increase glymphatic function and improve cerebral lymphatic clearance (Zhang F. et al., 2026). They described therapies which included pharmacological interventions targeting vascular endothelial growth factor C, photobiomodulation, and emerging lifestyle and surgical interventions. High intensity interval training has been shown to induce changes in astrocytes and increase clearance of CSF (Feng et al., 2023). A recently described surgical approach showed promising results in the treatment of Alzheimer’s disease by performing a lymphovenous anastomosis as a method to increase glymphatic function and cerebral lymphatic clearance (Li et al., 2024).

Another approach could involve therapies aimed at restoring olfactory function. Olfactory training would consist of repeated exposure to odorants over time with the intended neuroplastic effect of improving or remediating olfactory functioning. A systemic review of 18 articles has shown that olfactory training is associated with improved global cognition, and, in particular, verbal fluency and verbal learning/memory (Vance et al., 2024). In this review, olfactory training was also associated with increases in the volume/size of olfactory-related brain regions, including the olfactory bulb and hippocampus, and altered functional connectivity (Vance et al., 2024).

Cerebral blood flow (CBF) and olfactory sensing operate bidirectionally. Olfactory stimulation triggers regional CBF and increases olfactory processing areas, and, conversely, reduced CBF in key olfactory regions is associated with impaired smell (Kashibayashi et al., 2021). Cerebral blood flow is a critical driver of glymphatic function, with arterial pulsatility and neurovascular coupling directly propelling CSF through perivascular spaces to facilitate metabolic waste clearance from the brain. Improving or increasing cerebral blood flow would potentially increase the flow of toxic waste products through the glymphatic system (Holstein-Ronsbo et al., 2023).

NeuroEPO plus (NeuralCIM^®^) (a non-hematopoietic formulation of erythropoietin) has been primarily studied as a neuroprotective agent and has been shown to improve cognitive scores (Sosa et al., 2023). While the drug does not stimulate the production of new red blood cells or increase systemic blood volume, research suggests it supports brain health by improving impaired cerebral blood flow. More research is needed to investigate the use of drugs that will increase CBF to evaluate the impact on olfactory dysfunction, cognitive impairment, and sleep disturbances associated with neuropathological disorders.

Developing therapies aimed at directly reducing nasal turbinate vasodilation in patients with metabolic syndrome would be another approach based on the previously described findings by the authors (Phillips et al., 2022; Phillips and Schwartz, 2024). This type of intervention could interrupt the cascade of events (Figure 2) leading to the accumulation of toxic waste products in the olfactory regions of the brain. Future research directed toward reducing nasal turbinate vasodilation could possibly provide therapeutic benefits for the extensive number of neuropathological disorders associated with olfactory dysfunction.

## 15 Conclusion

Olfactory dysfunction represents a prevalent, early, and mechanistically informative manifestation of neuropathological disorders. The convergence of epidemiological, neuropathological, and neuroimaging evidence establishes olfactory dysfunction as a valuable biomarker for identifying individuals at risk for PD, AD, and related disorders. Standardized olfactory testing offers a practical, non-invasive approach for screening at-risk populations and enhancing early diagnosis. As the field advances toward precision medicine and disease-modifying therapies, integrating olfactory assessment into clinical practice and research protocols holds substantial promise for improving outcomes in conditions.

Although numerous mechanisms associated with neuropathological disease and olfactory dysfunction have been described in the literature, the initial event that triggers the development of any of these processes is not well understood. Based on their research, the authors of this article have speculated a triggering event, metabolic syndrome, which would lead to a cascade of predictable changes. Initiated by metabolic syndrome and glucose dysregulation, there is increased parasympathetic activity. The increase in parasympathetic activity causes nasal turbinate vessels to expand, placing pressure on and eventually obstructing nasal turbinate lymphatics. Obstruction of the lymphatic and CSF flow leads to accumulation of toxic waste which manifests in glymphatic dysfunction, and the development of neuropathological conditions.

The frequent detection of a unique cluster of manifestations, including glymphatic dysfunction, cognitive impairment, and sleep disturbances, in addition to olfactory dysfunction in various neuropathological disorders, suggests a common physiological etiology and warrants further investigation to promote novel therapeutic approaches.
